# Identification and Analysis of Estrogen Receptor *α* Promoting Tamoxifen Resistance-Related lncRNAs

**DOI:** 10.1155/2020/9031723

**Published:** 2020-04-28

**Authors:** Xiulei Zhang, Shanjun Gao, Zhen Li, Wei Wang, Guangzhi Liu

**Affiliations:** ^1^Department of Microbiome Laboratory, Henan Provincial People's Hospital, People's Hospital of Zhengzhou University, Zhengzhou, Henan 450003, China; ^2^Department of Obstetrics and Gynecology, Henan Provincial People's Hospital, People's Hospital of Zhengzhou University, Zhengzhou, Henan 450003, China

## Abstract

70-75% breast cancer patients are estrogen receptor alpha positive (ER*α*+), and the antiestrogen drug tamoxifen has been used for the past three decades. However, in 20-30% of these patients, tamoxifen therapy fails due to intrinsic or acquired resistance. A previous study has showed ER*α* signaling still exerts significant roles in the development of tamoxifen resistance and several lncRNAs have been demonstrated important roles in tamoxifen resistance. But ER*α* directly regulated and tamoxifen resistance related lncRNAs remain to be discovered. We reanalyze the published ER*α* chromatin immunoprecipitation-seq (ChIP-seq) and RNA-seq data of tamoxifen-sensitive (MCF-7/WT) and tamoxifen-resistant (MCF-7/TamR) breast cancer cells. We demonstrate that there are differential ER*α* recruitment events and the differentials may alert the expression profile in MCF-7/WT and MCF-7/TamR cells. Furthermore, we make an overlap of the ER*α* binding lncRNAs and differentially expressed lncRNAs and get 49 ER*α* positively regulated lncRNAs. Among these lncRNAs, the expression levels of AC117383.1, AC144450.1, RP11-15H20.6, and ATXN1-AS1 are negatively correlated with the survival probability of breast cancer patients and ELOVL2-AS1, PCOLCE-AS1, ITGA9-AS1, and FLNB-AS1 are positively correlated. These lncRNAs may be potential diagnosis or prognosis markers of tamoxifen resistance.

## 1. Introduction

Breast cancer is the most commonly diagnosed cancer and the leading cause of cancer death among female cancers, with an estimated 2.1 million newly diagnosed female breast cancer cases in 2018 around the world [1]. About 75% of all breast cancer cases are estrogen receptor alpha positive (ER*α*+); thus, the selective ER modulator tamoxifen has been the standard treatment for ER*α*+ breast cancer patients. However, up to 30% of these cases relapse due to tamoxifen resistance [2]. Various mechanisms have been proposed to explain tamoxifen resistance, including the abnormal ER*α* signaling pathway, alterations in cell cycle and apoptosis molecules, and activation of TGF-*β* or NF-*к*B signaling pathway [3, 4]. Noncoding RNAs (ncRNAs) also play an important role in tamoxifen resistance [5]. Long noncoding RNAs (lncRNAs) are a new type ncRNAs with more than 200 nucleotides, and they are involved in a wide variety of physiological and pathological processes [6]. What is more, lncRNAs have been demonstrated to play a significant role in drug resistance. Urothelial carcinoma associated 1 (UCA1) has been confirmed to contribute to multiple cancer drug resistance, including cisplatin resistance in bladder cancer and ovary cancer, gefitinib resistance in lung cancer, and tamoxifen resistance in breast cancer [7]. Besides, breast cancer antiestrogen resistance 4 (BCAR4), lncRNA-ROR (ROR, regulator of reprogramming), colon cancer associated transcript 2 (CCAT2), DSCAM-AS1, and LINC00894 are also reported to enhance or attenuate tamoxifen resistance [8]. But the important role and complex working mechanism of lncRNAs in tamoxifen resistance still need to be illustrated, and there is also an urgent need to discover more novel diagnosis and prognosis markers.

In this work, we reanalyze the published ER*α* ChIP-seq and RNA-seq data of tamoxifen-sensitive (MCF-7/WT) and tamoxifen-resistant (MCF-7/TamR) breast cancer cells [9]. We find that there are differential ER*α* recruitment events, and these events may lead to changes of expression profile between MCF-7/WT and MCF-7/TamR cells. And we also find that the ER*α* signaling is also estradiol (E2) dependent in MCF-7/TamR cells. Here, we provide insight into how ER*α* signaling promotes tamoxifen resistance through lncRNAs and get eight lncRNAs that may act as potential diagnosis or prognosis markers.

## 2. Materials and Methods

### 2.1. ChIP-seq and RNA-seq Data Analysis

The ChIP-seq and RNA-seq data were downloaded from the GEO dataset (GSE86538). Quality control of the raw reads was performed by FastQC [10]. ChIP-seq reads were aligned to the hg19 genome assembly using Bowtie 2 [11]. ChIP-seq peaks were called using MACS, and the *p* value 1.00*e*-05 was used as the cut-off [12]. RNA-seq reads were aligned and annotated using hg19 with Hisat2, and SAMtools was used to sort and reorder the SAM files [13, 14]. HT-seq and DESeq2 were used to analyze the differential expression, and fold change ≥ 2 or ≤0.5 and *p* value ≤ 0.05 were used as the cut-off [15, 16].

### 2.2. Visualization of the ChIP-seq Peaks and RNA-seq Differential Expression Genes

The aligned ChIP-seq reads were annotated and visualized using ChIPseeker, and (-3 kb_3 kb) was set as the transcription start site (TSS) region [17]. The RNA-seq differential expression genes (fold change ≥ 2 or ≤0.5, *p* value ≤ 0.05) were visualized as a heatmap using R package of ggplot2 [18].

### 2.3. Functional Enrichment Analysis

The ER*α* ChIP-seq binding genes in the (-3 kb_3 kb) region and upregulated genes in MCF-7/TamR cells (fold change ≥ 2, *p* value ≤ 0.05) were selected for the further functional enrichment analysis. We did the Gene Ontology (GO) and Kyoto Encyclopedia of Genes and Genomes (KEGG) enrichment analysis of the candidate genes using R package of ClusterProfiler, and *p* value ≤ 0.05 was used as the cut-off [19].

### 2.4. The Correlation Analysis and Survival Analysis

We analyzed the correlationship between expression level of candidate lncRNAs and ER*α* status using the breast invasive carcinoma (BRCA) data from The Cancer Genome Atlas (TCGA). We also analyzed whether the expression levels of candidate lncRNAs correlated with patient survival time (based on *p* value from the univariate Cox proportional hazards model and log-rank test) and visualized their correlationship through Kaplan-Meier plot. Spearman's rank correlation analysis was used to detect significant correlations between lncRNA and mRNA with a coefficient (absolute value) cut-off of 0.3 and a *p* value cut-off of 0.01. The correlation analysis and survival analysis were performed through the TANRIC website [20].

## 3. Results

### 3.1. The Differential ER*α* Recruitment Between MCF-7/WT and MCF-7/TamR Cells

**Figure 1:**
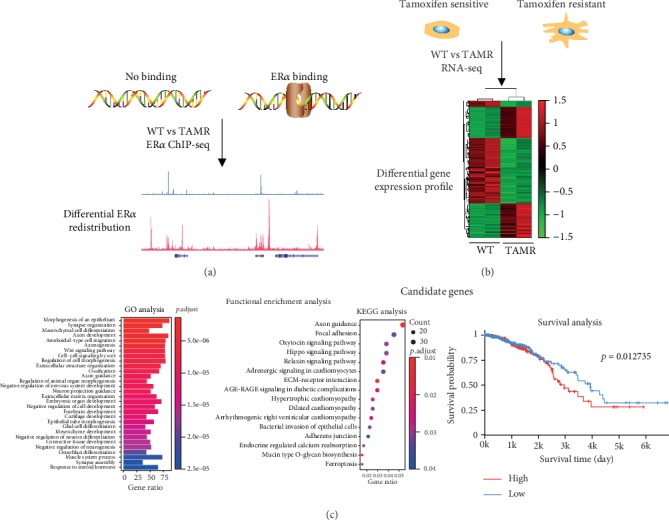
Proposed work flow of the procedure to identify the ER*α* promoting tamoxifen resistance-related lncRNAs. In breast cancer tamoxifen-sensitive cell MCF-7/WT (WT) and tamoxifen-resistant cell MCF-7/TamR (TAMR), (a) estrogen receptor *α* (ER*α*) binding or no binding events are determined by ChIP-seq and the differential ER*α* redistribution is visualized by UCSC genome browser; (b) the differential gene expression profile is investigated by RNA-seq and presented in a heatmap. (c) By analyzing the ChIP-seq and RNA-seq data from GSE86538, the overlap genes (candidate genes) may be ER*α* promoted and tamoxifen resistance related. And further, these genes will be verified by functional enrichment analysis by clusterProfiler package and survival analysis *via* the TANRIC website.

**Figure 2:**
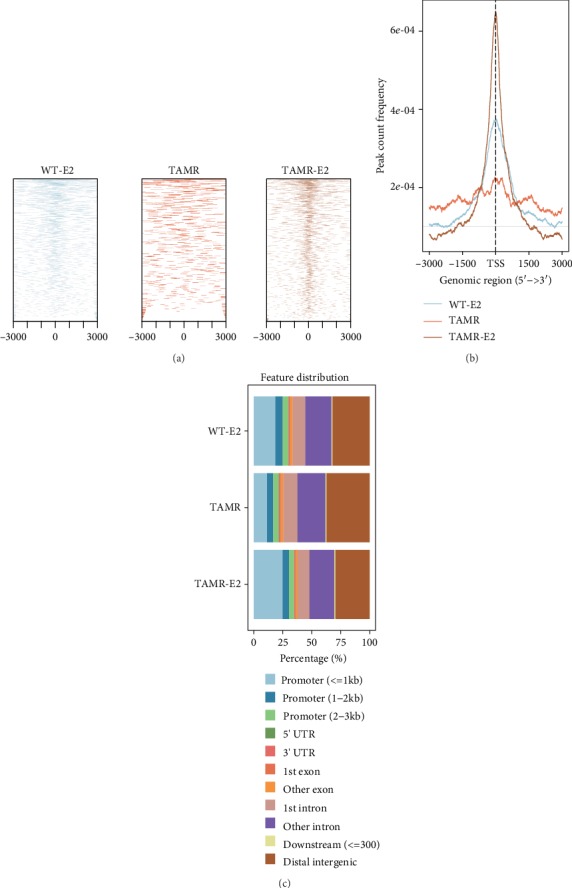
The distribution feature of ER*α* ChIP-seq peaks of MCF-7/WT with estradiol (E2) (WT-E2), MCF-7/TAMR without E2 (TAMR), and MCF-7/TAMR with E2 (TAMR-E2) cells. The profile (a) and peak count frequency (b) of ER*α* ChIP peaks binding to transcription start site (TSS) regions in WT-E2, TAMR, and TAMR-E2 cells. (c) Genomic annotation of ER*α* ChIP-seq peaks and their distribution percentage in different genomic region in WT-E2, TAMR, and TAMR-E2 cells.

**Figure 3:**
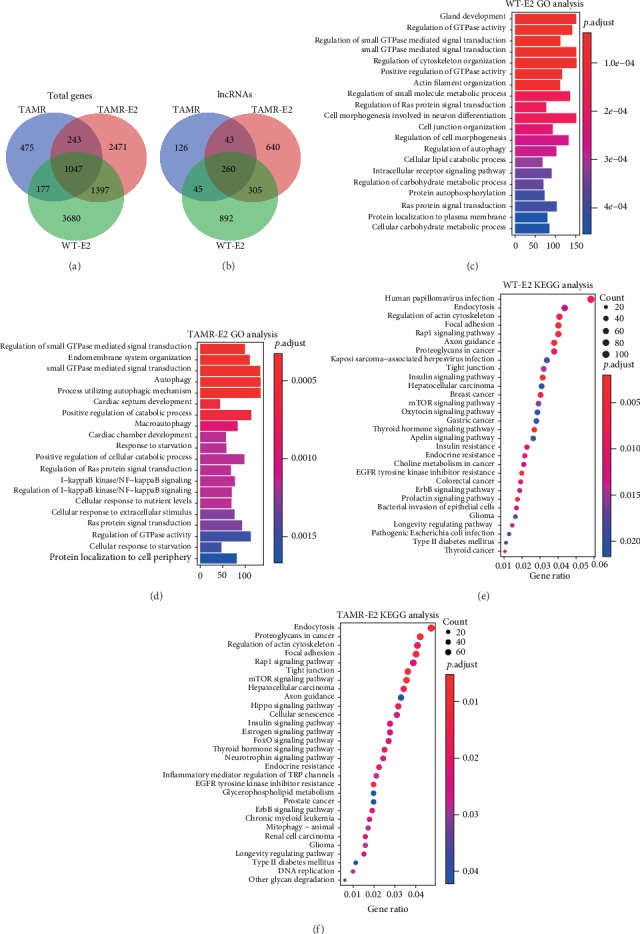
Functional enrichment analysis of ER*α* binding genes in MCF-7/WT-E2 and MCF-7/TamR-E2 cells. Venn plot of the overlap genes (a) or lncRNAs (b) of ER*α* binding genes (-3 kb_3 kb region) in MCF-7/WT-E2, MCF-7/TamR, and MCF-7/TamR-E2 cells. Bar plot of GO enrichment analysis of ER*α* binding genes (-3 kb_3 kb region) in MCF-7/WT-E2 (c) and MCF-7/TamR-E2 (d) cells. Dot plot of KEGG pathway enrichment analysis of ER*α* binding genes (-3 kb_3 kb region) in MCF-7/WT-E2 (e) and MCF-7/TamR-E2 (f) cells.

To determine whether there were differential ER*α* recruitment events between MCF-7/WT and MCF-7/TamR, we reanalyzed the published ER*α* ChIP-seq data of these cells (Figure 1(a)). The ER*α* binding peaks of MCF-7/WT and MCF-7/TamR cells that were stimulated with estradiol (E2) were more centralized around the transcription start site (TSS) (-3 kb_3 kb) than these peaks of MCF-7/TamR cells without E2 (Figures 2(a)–2(c)). What is more, MCF-7/TamR cells that were stimulated with E2 contained much more ER*α* binding peaks than these peaks of MCF-7/TamR cells without E2 (MCF-7/TamR-E2 17025 peaks, MCF-7/TamR 9703 peaks, *p* value ≤ 1.00*e*-05) (Table S1). The ER*α* directly regulated genes in MCF-7/TamR cells had 66.4% overlap of MCF-7/TamR-E2 cells (lncRNA, 63.9%). In contrast, the ER*α* directly regulated genes in MCF-7/TamR-E2 cells just had 25.0% overlap of MCF-7/TamR cells (lncRNA, 24.3%) (Figures 3(a) and 3(b)), which indicated that ER*α* signaling in MCF-7/TamR cells was also E2-dependent. GO enrichment analysis of ER*α* binding events showed that the misregulated autophagy and I-kB kinase/NF-kB signaling pathway in MCF-7/TamR cells may lead to tamoxifen resistance (Figures 3(c) and 3(d)). KEGG pathway enrichment analysis of ER*α* binding genes indicated that the cellular senescence, inflammatory mediator regulation of TRP channels, and mitophagy may regulate tamoxifen resistance (Figures 3(e) and 3(f)). The differential ER*α* recruitment events between MCF-7/WT and MCF-7/TamR cells may change their sensitivity to tamoxifen.

### 3.2. The Misregulated Genes in MCF-7/TamR Cells

**Figure 4:**
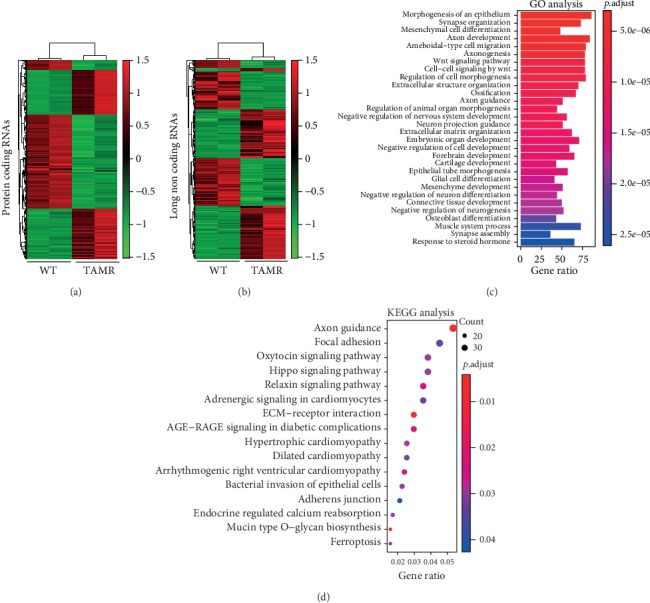
The misregulated genes and their functional enrichment analysis. Heatmap of misregulated protein-coding genes (a) and lncRNAs (b) in MCF-7/WT (WT) and MCF-7/TamR (TAMR) cells. Bar plot of GO enrichment analysis (c) and dot plot of KEGG pathway enrichment analysis (d) of upregulated genes in MCF-7/TamR cells (fold change ≥ 2, *p* value ≤ 0.05).

To find more misregulated genes in tamoxifen resistance, we further analyzed the published RNA-seq data of MCF-7/WT and MCF-7/TamR cells (Figure 1(b)). There were 4192 protein-coding genes (2006 upregulated, 2186 downregulated) and 1137 lncRNAs (588 upregulated, 549 downregulated) in MCF-7/TamR cells when compared with MCF-7/WT cells (fold change ≥ 2 or ≤0.5, *p* value ≤ 0.05) (Figures 4(a) and 4(b)) (Table S2). Many upregulated genes such as cancer stem cell marker (SOX2/9), hypoxia inducible factor (HIF1*α*), TGF-*β* signaling associated receptor (TGFBR2), and differentiation related transcription factor (RUNX2) were reported to promote tamoxifen resistance [21–24]. Functional annotation of the upregulated genes indicated that an enrichment of GO terms included mesenchymal cell differentiation, Wnt signaling pathway, extracellular structure organization, embryonic organ development, and response to steroid hormone (*p* ≤ 0.05) (Figure 4(c)). KEGG pathway analysis of the upregulated genes showed that an enrichment of signaling pathways included focal adhesion, Hippo signaling pathway, extracellular matrix-receptor interaction, adherens junction, and endocrine-regulated calcium reabsorption (*p* ≤ 0.05) (Figure 4(d)). These functions were consistent with the morphological changes and indicative of a metastatic phenotype that may promote tamoxifen resistance.

### 3.3. The ER*α* Directly and Positively Regulated lncRNAs

**Figure 5:**
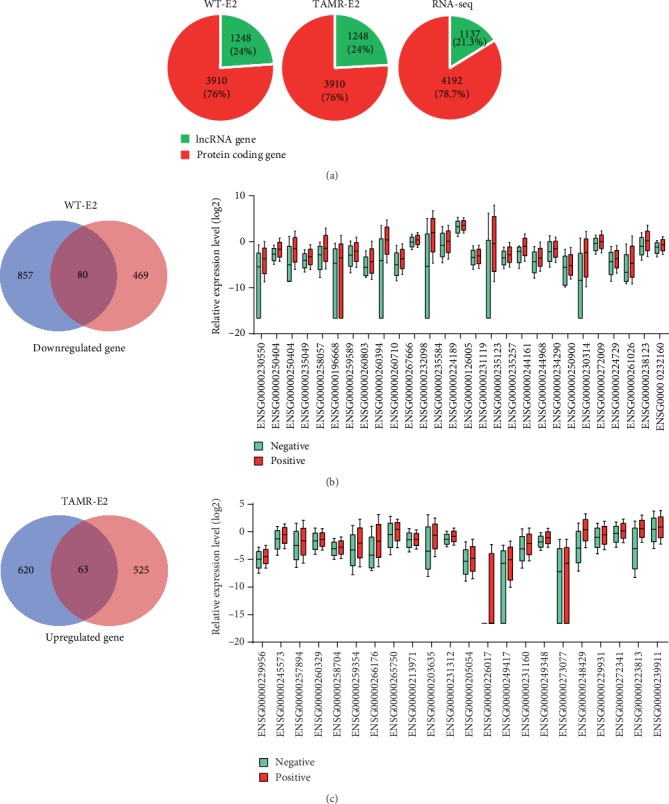
Identification of ER*α* directly promoting lncRNAs. (a) Pie graph of the percentage of protein-coding genes and lncRNAs of ER*α* binding genes (-3 kb_3 kb region) in MCF-7/WT-E2 (WT-E2) and MCF-7/TamR-E2 (TAMR-E2) cells and misregulated genes in MCF-7/TamR cells. (b) Venn plot of overlap lncRNAs of unique ER*α* binding lncRNAs (-3 kb_3 kb region) in MCF-7/WT-E2 (WT-E2) cells and downregulated lncRNAs in MCF-7/TamR cells and box plot of the relationship between relative expression level (log2) of overlap lncRNAs and the ER*α* status (positive or negative). (c) Venn plot of overlap lncRNAs of unique ER*α* binding lncRNAs (-3 kb_3 kb region) in MCF-7/TamR-E2 (TAMR-E2) cells and upregulated lncRNAs in MCF-7/TamR cells and box plot of the relationship between relative expression level (log2) of overlap lncRNAs and the ER*α* status.

The ER*α* recruitment events altered after the MCF-7/WT cells acquired tamoxifen resistance (Figures 2(a)–2(c)). The analysis of ER*α* ChIP-seq data of MCF-7/WT-E2 and MCF-7/TamR-E2 cells, respectively, showed 3857 and 2714 unique binding sites (937 lncRNA genes; 683 lncRNA genes) around TSS region (-3 kb_3 kb, *p* ≤ 1*e* − 5) (Figures 3(a) and 3(b)), and the redistribution of ER*α* binding may lead to the acquisition of tamoxifen resistance. The redistribution of *ERα* binding may change the expression profile in MCF-7/WT and MCF-7/TamR cells. The RNA-seq data of MCF-7/WT and MCF-7/TamR cells found 5329 genes (4192 protein-coding genes, 1137 lncRNA genes, fold change ≥ 2 or ≤0.5, *p* value ≤ 0.05) (Figure 5(a)). In order to find the ER*α* directly regulated genes in tamoxifen resistance, we analyzed the overlap genes between ER*α* binding genes and misregulated genes in MCF-7/TamR. We supposed that the lost ER*α* binding in MCF-7/TamR-E2 cells may result in downregulated genes and the acquired ER*α* binding may lead to upregulated genes. We found 80 overlap lncRNAs between unique lncRNAs in MCF-7/WT-E2 (ChIP-seq) and downregulated lncRNAs in MCF-7/TamR (RNA-seq) and 63 overlap lncRNAs between unique lncRNAs in MCF-7/TamR-E2 (ChIP-seq) and upregulated lncRNAs in MCF-7/TamR (RNA-seq) (Figures 5(b) and 5(c), left). We also analyzed the correlationship between the expression level of overlap lncRNAs and ER status (positive or negative) and got 49 candidate lncRNAs (Figures 5(b) and 5(c), right), which were directly and positively regulated by ER*α*.

### 3.4. Survival Analysis of the Candidate lncRNAs

**Figure 6:**
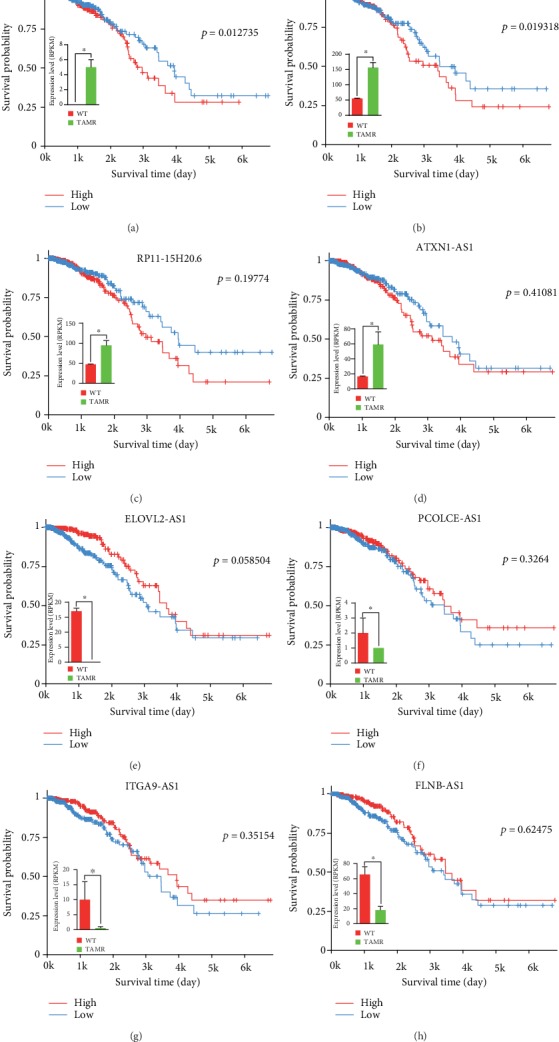
Survival analysis of the candidate lncRNAs. (a–h) Bar plot shows the expression level of the candidate lncRNAs in MCF-7/WT and MCF-7/TamR cells and Kaplan-Meier curve of the candidate lncRNAs expression and survival probability of breast cancer patients.

To verify whether the lncRNAs we identified in the above steps were associated with the prognosis, we analyzed the expression data of the lncRNAs and the clinical survival information to perform multivariate Cox regression *via* the TANRIC website. Four lncRNAs (AC117383.1, AC144450.1, RP11-15H20.6, and ATXN1-AS1) highly expressed in MCF-7/TamR cells were negatively related with the survival probability of breast cancer patients and other four lncRNAs (ELOVL2-AS1, PCOLCE-AS1, ITGA9-AS1, and FLNB-AS1) lowly expressed in MCF-7/TamR cells were positively related (Figures 6(a)–6(h)). To our knowledge, these eight lncRNAs have not been previously studied, and they may be promising prognosis markers of tamoxifen resistance.

### 3.5. Correlation Analysis between Candidate lncRNAs and mRNAs

By Spearman's rank correlation analysis between lncRNA and mRNA, we identified tens of significantly correlated mRNAs of the eight candidate lncRNAs (Table S3). Among the eight lncRNAs, there were five antisense lncRNAs (ATXN1-AS1, ELOVL2-AS1, PCOLCE-AS1, ITGA9-AS1, and FLNB-AS1). A previous study demonstrated that antisense lncRNA can hybridize with sense RNA and form RNA duplexes, which promoted the stability of the sense RNA [25]. In our results, we found that ATXN1-AS1, ELOVL2-AS1, and FLNB-AS1 were separately positively correlated with their sense RNA ATXN1, ELOVL2, and FLNB, which were essential genes in cellular activities. What is more, the rest five lncRNAs were also significantly correlated with key genes. AC117383.1 was positively correlated with ligand-dependent corepressor LCOR, AC144450.1, with ceramide synthase 6 LASS6, RP11-15H20.6 with zinc finger protein ZNF431, PCOLCE-AS1 with RNA-binding protein RBM5, and ITGA9-AS1 with C-terminal domain small phosphatase-like protein CTDSPL. The correlation analysis may demonstrate the important function of these eight lncRNAs and give us a clue to study their working mechanism.

## 4. Discussion

Tamoxifen therapy for five years reduced the annual breast cancer death rate by 34% and an absolute reduction in mortality of 9.2% at 15 years [2]. Despite that, there were 30% ER*α*+ breast cancer patients relapsed resulting from tamoxifen resistance [22]. In our work, we focused on ER*α*+ breast cancer, which accounted for three quarters of all breast cancer cases. We used the ER*α*+ breast cancer cells MCF-7/WT and its derived tamoxifen resistant cells MCF-7/TamR to investigate ER*α*-regulated and tamoxifen resistance-related lncRNAs. By analyzing the ER*α* ChIP-seq data of MCF-7/WT-E2 and MCF-7/TamR-E2 cells and RNA-seq data of MCF-7/WT and MCF-7/TamR cells, we may further understand the molecular mechanism of tamoxifen resistance and find more diagnosis and prognosis markers.

We supposed whether the ER*α* signaling in MCF-7/TamR cells was E2-dependent, and we analyzed the ER*α* ChIP-seq data of MCF-7/TamR cells without E2 stimulation and with E2 stimulation. We found that the ER*α* directly regulated genes in MCF-7/TamR cells had two-thirds overlap of MCF-7/TamR-E2 cells, and in contrast, the ER*α* directly regulated genes in MCF-7/TamR-E2 cells just had a quarter overlap of MCF-7/TamR cells, which indicated that ER*α* signaling in MCF-7/TamR cells was also E2-dependent. We wanted to find the differential signaling pathway that ER*α* regulated in MCF-7/WT and MCF-7/TamR cells. We did the GO enrichment analysis of the ER*α* binding genes and compared their differential functional annotation. A differential enrichment of GO terms included that autophagy and I-*κ*B kinase/NF-*κ*B signaling pathway, and these terms were demonstrated to take an important part in tamoxifen resistance [23, 24]. We proposed the upregulated lncRNAs in MCF-7/TamR cells may result from the ER*α* binding and the downregulated lncRNAs in MCF-7/TamR cells may due to the loss of ER*α* binding. So we analyzed the RNA-seq data of MCF-7/WT and MCF-7/TamR cells and made an overlap of the ER*α* binding genes and misregulated genes in MCF-7/TamR cells. But the overlap just made up 5.69% and 5.22% of these genes, which meant other signaling pathways except ER*α* signaling also played an important role in tamoxifen resistance. Despite that, we demonstrated the positive relation between the ER*α* status and the 143 overlap lncRNAs by analyzing the TCGA data [20]. These above steps and further survival analysis helped us get eight candidate lncRNAs. Furthermore, we did Spearman's rank correlation analysis of these eight lncRNAs and may demonstrate their important function in cellular activities. This bioinformatics process facilitated the selection of most possible tamoxifen resistance-related lncRNAs.

To our knowledge, these eight lncRNAs have not been previously studied, and they may be promising tamoxifen resistance diagnosis or prognosis markers. Further understanding the function of the eight lncRNAs will help the clinician to early diagnose whether the patients are tamoxifen resistant and bring some clinical indications in the development of novel prognostic factors in breast cancer.

## 5. Conclusion

In this study, we show the differential ER distribution events and differential expression profile in tamoxifen-sensitive and tamoxifen-resistant breast cancer cells and further demonstrate that the ER signaling pathway in tamoxifen-resistant breast cancer cells is also E2-dependent. Besides, we find eight candidate lncRNAs that may serve as diagnosis or prognosis markers.

## Data Availability

The data used to support the findings of this study are included within the article.

## Abbreviations

ER*α*+: Estrogen receptor alpha positive
lncRNA: Long noncoding RNA
ChIP-seq: Chromatin immunoprecipitation-seq
MCF-7/WT: MCF-7 tamoxifen-sensitive cell line
MCF-7/TamR: MCF-7-derived tamoxifen-resistant cell line
MCF-7/WT-E2: MCF-7/WT cell with estradiol (E2) stimulation
MCF-7/TamR-E2: MCF-7/TamR cell with estradiol (E2) stimulation.

## Supplementary Materials

Supplementary 1 Table S1: ER binding peaks in MCF-7/TamR and MCF-7/TamR-E2 cells.

Supplementary 2 Table S2: differentially expressed protein-coding genes and lncRNAs.

Supplementary 3 Table S3: correlation analysis between candidate lncRNAs and mRNAs.
